# Spinal-generated movement disorders: a clinical review

**DOI:** 10.1186/s40734-015-0028-1

**Published:** 2015-12-24

**Authors:** Pichet Termsarasab, Thananan Thammongkolchai, Steven J. Frucht

**Affiliations:** Department of Neurology, Movement Disorder Division, Icahn School of Medicine at Mount Sinai, New York, USA; Department of Medicine, Neurology Division, Faculty of Medicine, Siriraj Hospital, Mahidol University, Bangkok, Thailand; Department of Neurology, University Hospitals Case Medical Center, Cleveland, USA

**Keywords:** Spinal cord, Spinal myoclonus, Orthostatic tremor, Paroxysmal dyskinesia, Stiff person syndrome, Brain death, Painful legs-moving toes syndrome

## Abstract

Spinal-generated movement disorders (SGMDs) include spinal segmental myoclonus, propriospinal myoclonus, orthostatic tremor, secondary paroxysmal dyskinesias, stiff person syndrome and its variants, movements in brain death, and painful legs-moving toes syndrome. In this paper, we review the relevant anatomy and physiology of SGMDs, characterize and demonstrate their clinical features, and present a practical approach to the diagnosis and management of these unusual disorders.

**Electronic supplementary material:**

The online version of this article (doi:10.1186/s40734-015-0028-1) contains supplementary material, which is available to authorized users.

## Introduction

Spinal-generated movement disorders (SGMDs) refers to those movement disorders that originate from the spinal cord, or in which the spinal cord plays an important role. SGMDs are central in origin, although in some SGMDs (such as painful legs-moving toes syndrome (PLMT) or orthostatic tremor (OT)), both peripheral and supraspinal mechanisms are also likely involved. We chose to highlight SGMDs in this review because they are unusual, and by considering them as a group we may highlight similar approaches to evaluation and treatment [1]. Before discussing SGMDs, we will review the relevant anatomy of the spinal cord.

## Review

### Spinal cord anatomy and physiology

**Fig. 1:**
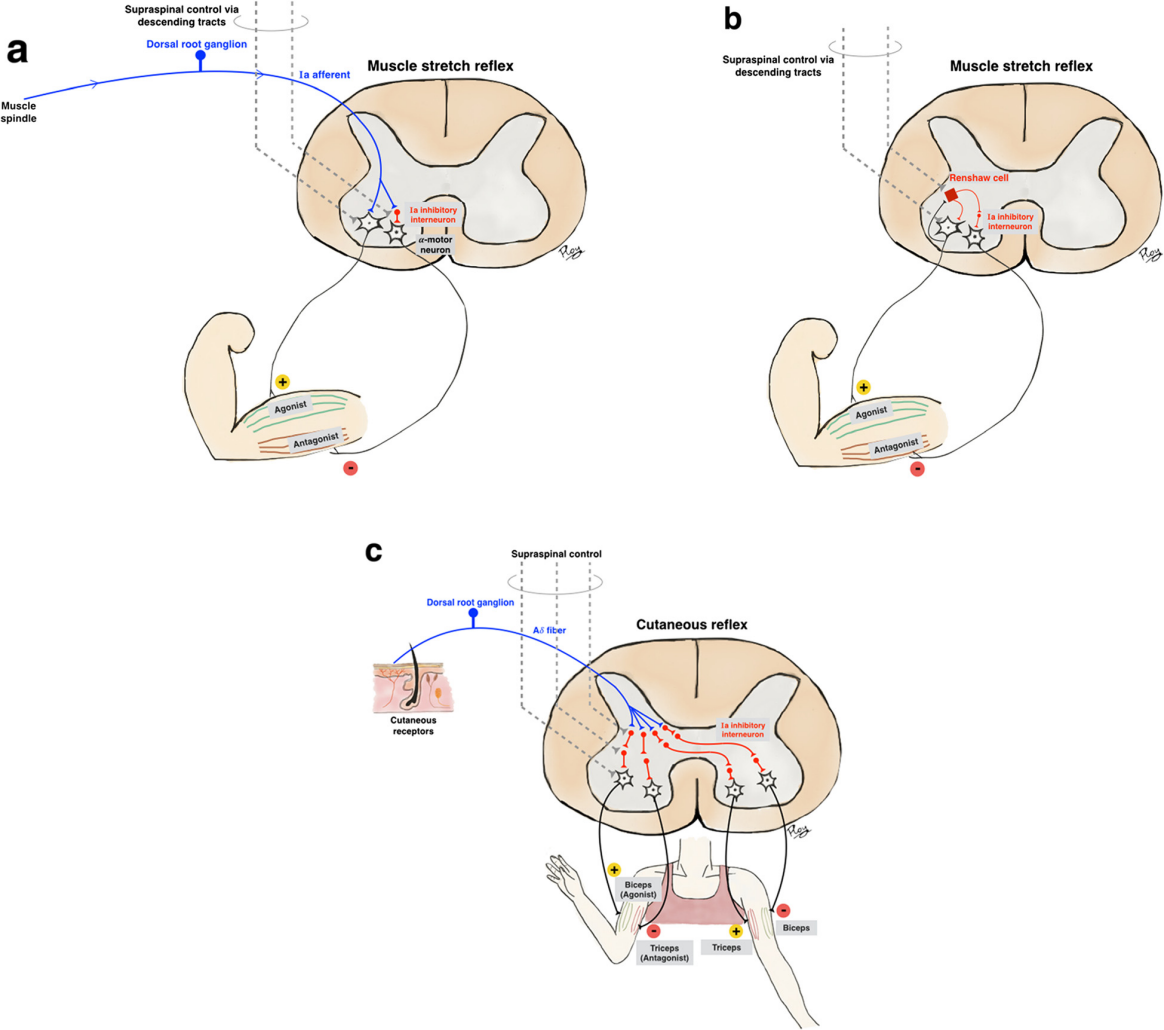
Major spinal reflexes include muscle stretch and cutaneous reflexes. **a**. The muscle stretch reflex. The afferent signal of the muscle stretch reflex from the muscle spindle is conveyed via Ia afferent fibers which are axons of dorsal root ganglia. The proximal axons then synapse directly with the alpha-motor neuron (a monosynaptic reflex), which in turn conveys an efferent signal to the corresponding extrafusal fibers of the agonist muscle leading to muscle contraction. Ia afferent fibers also synapse with Ia inhibitory interneurons, which in turn convey inhibitory signals to antagonist muscles, (reciprocal inhibition). **b**. The feedback control of the muscle stretch reflex. The Renshaw cell is the specialized inhibitory interneuron that functions in feedback control of the alpha-motor neurons in A. It receives input from collateral axons of the alpha-motor neuron controlling agonist muscles, and sends inhibitory signals back to the same alpha-motor neuron and also inhibitory signals to the alpha-motor neuron controlling antagonist muscles. The alpha-motor neurons, inhibitory interneurons and Renshaw cells also receive supraspinal control via descending tracts such as corticospinal or rubrospinal pathways. **c**. The cutaneous or flexion-withdrawal reflex. The cutaneous or flexion-withdrawal reflex is a polysynaptic reflex. Cutaneous nociceptive receptors send afferent signals via Aδ fibers which synapse with multiple interneurons before finally synapsing with the alpha-motor neuron. The interneurons connect the afferent and efferent signals, resulting in an excitatory signal to the ipsilateral flexor and contralateral extensor muscles, and an inhibitory signal to the ipsilateral extensor and contralateral flexor muscles. By this mechanism, flexion of the ipsilateral agonist muscle withdraws the limb from the nociceptive stimuli. An opposite chain is reversed in the opposite limb to prepare for support

Afferent signals are conveyed from the axons of dorsal root ganglia to the spinal cord gray matter. Efferent signals originate in the anterior (ventral) horn cells or alpha-motor neurons. Relay circuits may be monosynaptic (such as the muscle stretch reflex), or polysynaptic, (such as the flexion-withdrawal reflex). In the muscle stretch reflex, the afferent signal comes from the muscle spindle via Ia afferent fibers, and an efferent signal is conveyed to the corresponding extrafusal muscle fiber, leading to muscle contraction (Fig. 1a). Ia afferent fibers also convey signals to Ia inhibitory interneurons to inhibit the antagonist muscle, a phenomenon called reciprocal inhibition (Fig. 1a). The Renshaw cell, another type of spinal interneuron, functions in the feedback system by receiving the signal from the collateral axons of the alpha-motor neuron and sending an inhibitory signal to the same alpha-motor neuron and Ia inhibitory interneuron (Fig. 1b).

The second major reflex loop is the flexion-withdrawal reflex. Cutaneous nociceptive receptors send afferent signals via Aδ fibers which synapse with multiple interneurons before finally synapsing with the alpha-motor neuron (Fig. 1c). The final output is excitation and inhibition (respectively) of the ipsilateral flexor and extensor muscles, and vice versa on the contralateral side. The end result is an ipsilateral limb withdrawal from the nociceptive stimuli, while the opposite limb prepares for support. In another reflex loop, Golgi tendon organs via Ib afferent fibers, along with joint and cutaneous receptors, convey signals to Ib inhibitory interneurons (not shown).

Spinal interneurons receive supraspinal descending influences from the motor cortex, brainstem and cerebellum via descending pathways (including corticospinal and rubrospinal pathways), helping to integrate and modulate movement. The principal neurotransmitters of spinal interneurons are gamma-aminobutyric acid (GABA) and glycine. When spinal inhibitory interneuron activity is reduced, for example due to impaired supraspinal control, alpha-motor neurons become hyperexcitable leading to abnormal involuntary movements.

Control of locomotion is extremely complex, and a full discussion of spinal control of gait is beyond the scope of this manuscript. We only briefly touch on several relevant mechanisms. The locomotor stepping pattern, the complex sequence of activation of flexor and extensor muscles required for walking, is controlled by the spinal locomotor system. In humans, both afferent sensory signals from peripheral receptors and central pattern generators (groups of neurons within the spinal cord that can generate rhythmic motor activities without sensory afferent input [2, 3]), play an important role in locomotion. Supraspinal control from the mesencephalic locomotor region and the cerebellum also modulates the spinal locomotor system. Propriospinal pathways ascend and descend along the long axis of the spinal cord to interconnect short and long segments. Their presence and role in humans has not yet been proven, but likely they help to coordinate forelimb and hindlimb movements during locomotion in quadrupeds [4].

**Fig. 2:**
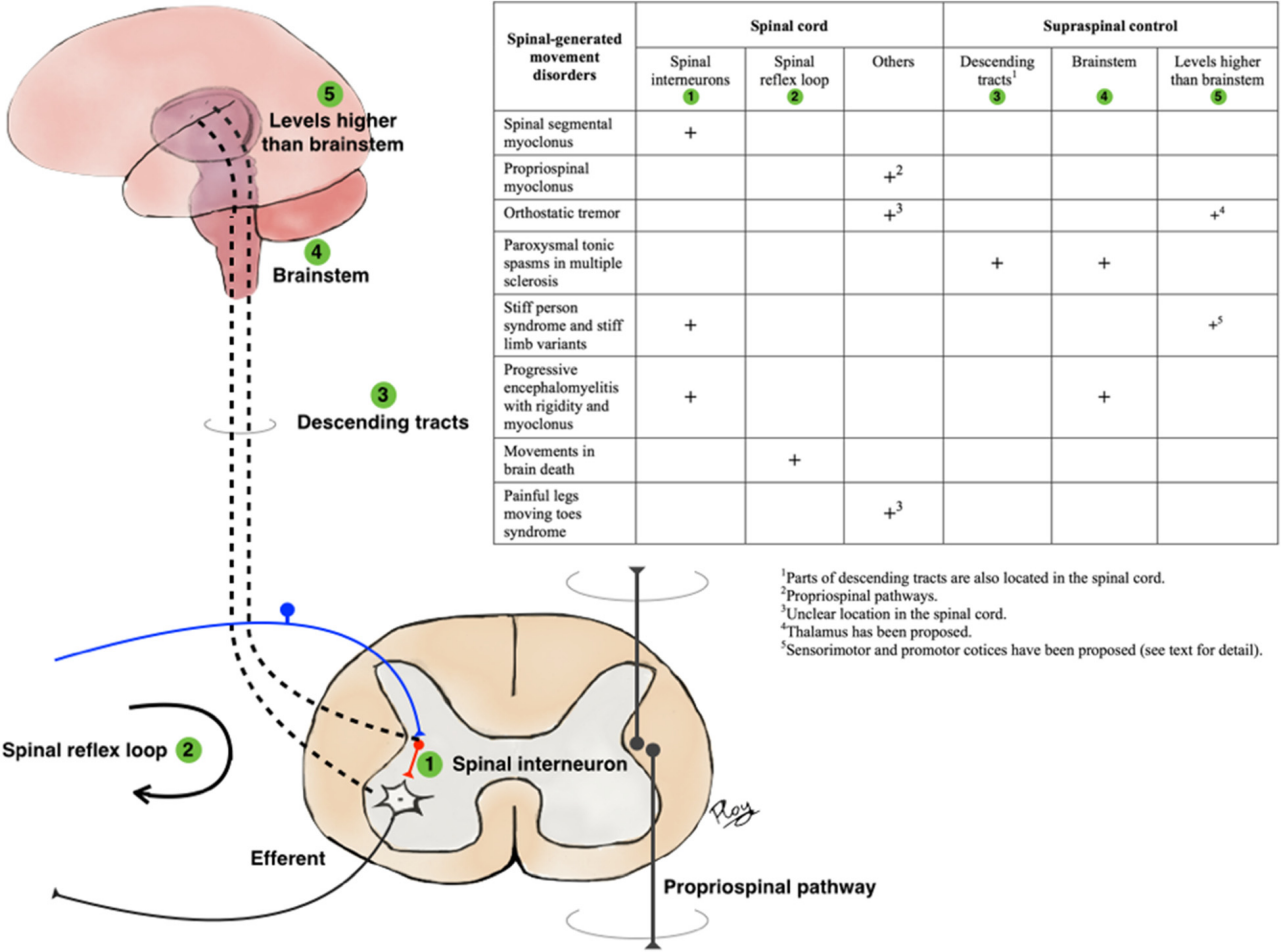
Localization in SGMDs. The figure illustrates the anatomical localization from the spinal cord, including the spinal reflex loop with inhibitory interneurons. The propriospinal pathway is shown on the opposite side. The interneurons and alpha-motor neuron also receives supraspinal control via the descending tracts. Higher levels of control including brainstem and thalamus are also depicted. The table demonstrates the location(s) that is(are) responsible for the pathophysiology in each SGMD. The location is classified as 1) the spinal cord including spinal interneurons, spinal reflex loops and other spinal locations, and 2) supraspinal control including descending tracts (part of which is also located in the spinal cord), brainstem and others

Knowledge of spinal cord physiology is useful in understanding the pathophysiology of SGMDs. For example, dysfunction of inhibitory interneurons leads to spinal segmental myoclonus. In stiff person syndrome, reduced input of inhibitory neurons via supraspinal control has been implicated. Automatic stepping patterns in near-brain death may occur due to disconnection of supraspinal control, allowing the central pattern generators to fire uninhibited. A summary of lesions and their location responsible for SGMDs is discussed further in the text below and illustrated in Fig. 2.

#### General considerations

If the clinician suspects that the spinal cord is a possible generator of involuntary movements (i.e., anatomically localizes the movements based on the distribution), phenomenology (i.e., classifying the movements based on their characters) may be useful in diagnosis. When myoclonic jerks are present, spinal segmental myoclonus (SSM), propriospinal myoclonus (PSM), and progressive encephalomyelitis with rigidity and myoclonus (PERM) should be considered. Orthostatic tremor (OT) is an example of a SGMD with tremor. Dystonic posturing is a phenomenon in secondary paroxysmal dyskinesias, including paroxysmal tonic spasms in multiple sclerosis (MS). SGMDs with stiffness include stiff person syndrome (SPS) and its variants, stiff limb syndrome (SLS) and PERM. Movements in brain death are often stereotyped, similar to primitive spinal reflexes or stepping patterns.

Certain examination findings may also help guide the diagnosis. One common pattern in SGMDs is involvement of one or multiple limbs without axial or bulbar involvement. Monocrural involvement occurs in SSM, SLS, and PLMT. Less commonly these disorders may affect two limbs. Almost all SGMDs lack bulbar or cranial involvement with few exceptions such as PERM, some cases of paroxysmal tonic movements in MS and undulating facial movements in brain death. Most SGMDs are typically hyperkinetic: myoclonus, tremor, dystonia, spinal reflexes or locomotor stepping movements all involve excess movements. Stiffness syndromes such as SPS or SLS may appear hypokinetic, but are better classified as sustained hyperkinetic spasms.

We next review the main clinical features, pathophysiology, and treatment of each SGMD (see Table 1 for summary). While treatment of SGMDs may include multiple modalities, pharmacotherapy of each SGMD is summarized in Table 2.

**Table 1:** Summary of phenomenology, pathophysiology, main clinical features, investigations and treatment of spinal-generated movement disorders (SGMDs)

SGMDs	Phenomenology	Pathophysiology	Main clinical features	Investigations	Treatment
Spinal segmental myoclonus (SSM)	Myoclonus	• Loss of inhibition of spinal interneurons → hyperexcitation of anterior horn cells	• Jerks of 1 or 2 limbs • Rhythmic or semi-rhythmic • Generally not stimulus-sensitive	• MRI of the spinal cord	• Rx of specific etiologies. • CLZ, VPA or LVT for symptomatic Rx
Propriospinal myoclonus (PSM)	Myoclonus	• Possible defects in propriospinal pathways (not yet proven in humans) • Psychogenic etiology also proposed	• Slow truncal jerking • Flexion more common than extension • Stimulus-sensitive, but longer latency than cortical myoclonus	• MRI of the spinal cord • EP testing	• Rx of specific etiologies in 2° forms • CLZ or VPA for symptomatic Rx
Orthostatic tremor (OT)	Tremor	• Unclear • Proposed tremor generators: brainstem, thalamus, and spinal cord • 1° and 2° (OT-plus) forms exist	• 13–18 Hz; in legs and trunk • Present when standing but not walking • Subjective unsteadiness; tremor may not be visible • Improved when lightly touching a table or wall • “Helicopter sign”	• EP testing • MRI of the brain or spinal cord (if exam is abnormal and 2° OT is suspected)	• Mainly CLZ or GBP • L-dopa or DA may be used in cases with concomitant parkinsonism
Paroxysmal tonic spasms in multiple sclerosis (MS) and neuromyelitis optica spectrum disorders (NMOSD)	Dystonia	• Ephaptic transmission between partially-demyelinated axons anywhere in central nervous system • Common locations: contralateral cerebral peduncle, internal capsule, and spinal cord	• Painful; involves unilateral arm or leg • Typically non-kinesigenic • Last several seconds to minutes • May involve ipsilateral face • Aggravated by hyperventilation • Can be initial presentation of MS	• MRI of the brain and spinal cord • Other MS/NMOSD work-up including CSF studies	• CBZ or acetazolamide
Stiff person syndrome (SPS) and its variants	Stiffness	• Impaired spinal GABAergic and glycinergic inhibitory circuits → CMUA • Supraspinal mechanisms proposed	• *Classic form*: stiffness of the trunk and legs, hyperlordotic gait • *Stiff limb variant*: involves only 1–2 limbs	• Ab testing (serum anti-GAD, anti-amphiphysin; less commonly anti-glycine or GABA_A_ receptor) • Malignancy work-up	• Rx of specific etiology if any • Immunosuppressive Rx: steroid, IVIg, and/or PLEX; chronic oral agents such as MMF, AZA and CYC • BZDs for symptomatic Rx
Progressive encephalomyelitis with rigidity and myoclonus (a variant of SPS)	Stiffness, myoclonus	• Loss of spinal inhibitory interneurons • Brainstem also involved	• Myoclonic jerks of the trunk, limbs and cranial muscles • +/− Nystagmus, oculomotor abnormalities, dysarthria and dysphagia	• Ab testing (serum and CSF anti-GAD, anti-glycine receptor, anti-DPPX) • MRI of the spinal cord and brainstem • CSF studies may be required	• Rx of specific etiology if any • Immunosuppressive Rx as in SPS
Movements in brain death and automatic stepping*	Spinal reflexes	• Disconnection of supraspinal control → disinhibition of the spinal reflexes • Some proposed released phylogenetically primitive patterns • Automatic stepping: spinal automatism from spinal CPG	• 2 types (examples shown) - *Automatisms* (abdominal contraction, undulating toe movements) - *Reflexes* (after neck flexion, finger pinching or testing Babinski sign) • Automatic stepping reported in a near brain-dead patient	• Confirmation of brain death (physical exam, apnea testing or TCD, etc.)	• Family reassurance
Painful legs-moving toes syndrome (PLMT)	Miscellaneous	• Unknown • Proposed mechanism: peripheral nerve pathology → 2° impairment in spinal and/or supraspinal controls, and central sensitization	• Slow 1–2 Hz, athetoid-like • Involves fingers or toes • Moves in vertical and/or horizontal planes • Pain usually the most debilitating symptom • Painless form present	• Work-up for associated neuropathies or radiculopathies depending on clinical context • MRI of the spinal cord rarely required	• Rx of concomitant diseases such as neuropathies • GBP or PGB • Others (case reports): baclofen, CBZ, BZD, TCA, SCS, epidural block, sympathetic blockade, TENS, BoNT

Summary table of pathophysiology, clinical features, investigations and treatment of SGMDs

*Movements in brain death and automatic stepping are normal findings, not "disorders".

*Abbreviations*: *MRI* magnetic resonance imaging, *Rx* treatment, *CLZ* clonazepam, *VPA* valproate, *LVT* levetiracetam, *EP* electrophysiologic,*1°* primary, *2°* secondary, *GBP* gabapentin, *L-dopa* levodopa, *DA* dopamine agonist, *CBZ* carbamazepine, *CSF* cerebrospinal fluid, *GABA* gamma-aminobutyric acid, *CMUA* continuous motor unit activity, *Ab* antibody, *GAD* glutamic acid decarboxylase, *IVIg* intravenous immunoglobulin, *PLEX* plasma exchange, *MMF* mycophenolate mofetil, *AZA* azathioprine, *CYC* cyclophosphamide, *BZD* benzodiazepine, *CPG* spinal central pattern generator, *TCD* transcranial Doppler ultrasound, *PGB* pregabalin, *TCA* tricyclic antidepressants, *SCS* spinal cord stimulation, *TENS* transcutaneous electrical nerve stimulation, *BoNT*, botulinum toxin injection

**Table 2:** Pharmacological treatment of each spinal-generated movement disorder

SGMDs	CLZ	VPA	LVT	CBZ	GBP	ImRx^a^	Others
Spinal segmental myoclonus (SSM)	+	+	+				
Propriospinal myoclonus (PSM)	+	+					
Orthostatic tremor (OT)	+				+		+^b^
Paroxysmal tonic spasms in multiple sclerosis (MS)				+			+^c^
Stiff person syndrome (SPS) and its variants	+/−^d^					+	+^e^
Progressive encephalomyelitis with rigidity and myoclonus (a variant of SPS)	+/−^d^					+	+^e^
Painful legs-moving toes syndrome (PLMT)	+^f^			+^f^	+		+^g^

Pharmacologic therapies in SGMDs. The effective or possibly effective therapies are indicated by “+”. Drugs utilized in SGMDs are mostly antiepileptics (including benzodiazepines, especially clonazepam, valproate, levetiracetam, carbamazepine, and gabapentin), and immunotherapies^a^ (including steroids, intravenous immunoglobulin, and/or plasma exchange, as well as immunosuppressants such as azathioprine, cyclophosphamide and mycophenolate mofetil)

*Abbreviations*: *SGMDs* spinal-generated movement disorders, *CLZ* clonazepam, *VPA* valproate, *LVT* levetiracetam, *CBZ* carbamazepine, *GBP* gabapentin, *ImRx* immunotherapies

^b^Dopaminergic therapies including dopamine agonists and levodopa in case with co-existing parkinsonism

^c^Acetazolamide

^d^Clonazepam may be used, but anecdotally is less effective than diazepam

^e^Benzodiazepines, especially diazepam

^f^In our experience, these medications are used less often than gabapentin

^g^Pregabalin is also used. Other medications reported in small number of patients include baclofen, carbamazepine, and tricyclic antidepressants

### Spinal segmental myoclonus (SSM)

SSM originates from one or a few adjacent segments of the spinal cord. Loss of inhibition of spinal interneurons leads to hyperexcitation of anterior horn cells [5]. SSM is typically rhythmic or semi-rhythmic, approximately 1–2 Hz in frequency, usually stimulus-*insensitive* [6], and involves one (or less commonly two) limbs or truncal/abdominal muscles (Additional file 1: Video segment 1). While there is no systematic study in the literature on the frequency of SSM among different spinal segments or myotomes, the involved body part(s) is(are) likely correlated with the anatomical location of the lesion(s) within the spinal cord. Differentiating SSM from myoclonus originating from the periphery (nerve roots, plexus or peripheral nerves) may be challenging. One important clue is that in peripherally-generated myoclonus, the distribution of muscles involved corresponds to the nerve roots [7, 8], plexus [9] or peripheral nerves [10–13], whereas SSM typically involves multiple muscles innervated by one to three adjacent spinal levels and can be bilateral. In SSM patients it is critical to exclude structural lesions of the spinal cord such as vascular, inflammatory [14, 15], infectious [16, 17], postinfectious [18], demyelinating [19], degenerative [20, 21], paraneoplastic [22], and space-occupying lesions [23, 24] with contrast MRI imaging. Specific treatment addresses the underlying etiology if one is found, while symptomatic treatments include clonazepam [21, 25, 26], valproic acid [26] and levetiracetam [27].

### Propriospinal myoclonus (PSM)

PSM may originate from the propriospinal pathways, which are classically distinguished by their relatively slow conduction in contrast to the faster-conducting corticospinal pathways. However, the presence and role of propriospinal pathways in humans has not yet been confirmed. Axial musculature is purely or predominantly involved in PSM, and patients typically have slow flexion truncal jerks that may be stimulus-sensitive (for example in response to tactile stimuli) (Additional file 1: Video segment 1). The latency between the examiner eliciting the stimulus and the patient’s movement is noticeably longer than in cortical myoclonus. This delay in onset of a truncal jerk after eliciting a reflex can be very helpful in the office evaluation of these patients, as demonstrated in Additional file 1: Video segment 1. Electrophysiology typically reveals recruitment or simultaneous spread of ascending and descending segments from a thoracic spinal source. PSM may be idiopathic or may be associated with spinal cord lesions. Some authors considered propriospinal myoclonus to be a functional disorder, due to the finding of Bereitschaftspotential or pre-movement potentials in some PSM patients [28–32]. Distinguishing between organic and psychogenic PSM based on clinical grounds alone may not be reliable, and there is thus a role of electrophysiologic study [28, 29, 32]. However, a recent study found true microstructural defects such as disruption of fiber tracts in the spinal cord using diffusion tensor imaging [33]. Our personal view, supported by van der Salm et al. [28], is that not all PSM cases are psychogenic: there are both organic and psychogenic forms. Given similar clinical features among patients and that several movement disorders such as dystonia was, in history, incorrectly envisioned as a psychogenic disorder due to inadequate knowledge in pathophysiology, the strong conclusion on psychogenicity to the whole group of PSM patients has to be made with caution.

### Orthostatic tremor (OT)

OT is a very fine fast tremor, felt in the legs when a patient stands, classically disappearing when the patient walks [34]. Patients typically complain of unsteadiness, and if symptoms have been present for a prolonged period they may complain of inability to walk or gait fearfulness (Additional file 2: Video segment 2). The oscillations may be audible when a stethoscope is placed behind the knee, producing a characteristic sound (“the helicopter sign”) [35]. OT is one of the highest frequency tremors, (13–18 Hz) [36, 37], and tremor may be invisible on exam [38, 39] but is usually palpable. In patients with no visible tremor, the diagnosis can generally be made by the history and examination illustrating inability to stand or unsteadiness while standing with improvement or no difficulty when walking. Tremor may also involve axial musculature or even the arms in the weight-bearing position [40]. Interestingly, tremor may be immediately attenuated by lightly touching objects such as a table, a wall, or the examiner’s hand (Additional file 2: Video segment 2). An important differential in OT is orthostatic myoclonus (OM), usually distinguished by electrophysiology [41, 42].

OT may be primary/idiopathic or secondary (or OT-plus), the later accompanying pontine [43], spinal [44], cerebellar [45], or parkinsonian pathology [46]. Two cases of OT with concomitant hydrocephalus in the setting of aqueductal stenosis and chronic relapsing polyradiculoneuropathy have been reported [47]. Investigations such as neuroimaging of the brain or spinal cord in OT patients are not mandated unless other neurologic signs are present. OT is typically treated with clonazepam or gabapentin [48], and response to treatment can occasionally be dramatic (Additional file 2: Video segment 2). Dopamine agonists or levodopa may be useful, especially in cases with concomitant parkinsonism [46, 49]. Other medications such as propranolol, primidone, phenytoin, carbamazepine and levetiracetam have been used, with variable success [46, 50]. Possible sources of the generator in OT include supraspinal structures such as the brainstem [51, 52], thalamus [53], spinal cord [52, 54] and cerebellum [55]. However, the emergence of OT after spinal cord lesions [54], and the benefit of spinal cord stimulation in medically-refractory OT [56] support a possible spinal etiology.

As a mimicker of OT, OM will be discussed briefly here. Patients with OM usually present with gait unsteadiness and a complaint of leg shaking or jerking [41, 42]. Difficulty initiating gait or “gait apraxia” is often found [41, 42]. Due to these symptoms, patients may be misdiagnosed as normal pressure hydrocephalus or OT [42]. Over 50–70 % of OM patients in two studies had associated neurodegenerative diseases including Parkinson’s disease, dementia with Lewy bodies, multiple system atrophy and Alzheimer’s disease [41, 42]. In addition to OT, it is thought to be one of under-recognized causes of unsteadiness [57, 58], and the diagnosis is confirmed by electrophysiologic study demonstrating myoclonus. Treatments that have been used in OM include clonazepam [42] and levetiracetam with variable results [42, 57]. However, the strong evidence is still lacking due to a small number of patients and short follow up duration.

### Secondary paroxysmal dyskinesias, including paroxysmal tonic spasms in multiple sclerosis (MS) and neuromyelitis optica spectrum disorders (NMOSD)

Secondary paroxysmal dyskinesias may involve the spinal cord. Important clues that a paroxysmal dyskinesia is secondary rather than primary include the presence of significant pain [59] and an abnormal interictal examination. Secondary paroxysmal dyskinesias may occur in hypoparathyroidism [60], pseudohypoparathyroidism [61, 62], supraspinal lesions [59, 63], spinal cord glioma [64] and spinal cord compression [65, 66].

Paroxysmal tonic spasms in MS, also called paroxysmal dystonia or secondary dyskinesias, are one of the most common causes of secondary paroxysmal dyskinesias. Painful dystonic spasms usually involve an ipsilateral arm and/or leg or face in some cases, without kinesigenic trigger, lasting several seconds to minutes [67, 68]. Episodes are often aggravated by hyperventilation, likely due to respiratory alkalosis and increased ephaptic transmission between partially demyelinated axons [69]. Paroxysmal tonic spasms can occur as the initial presentation of MS [70], but are usually seen once the disease is well established. It is important to remember that paroxysmal spasms of MS are not diagnostic of a spinal cord locus, and they may also originate in the contralateral cerebral peduncle [71], thalamus [72, 73], or internal capsule [71, 74, 75]. Paroxysmal tonic spasms often behave independently of MS; they can remit in spite of progression of MS or vice versa, and an EEG may be required to rule out seizures or epilepsia partialis continua [76, 77]. Paroxysmal tonic spasms are even more common in NMOSD than in MS [78, 79]. One recent study in NMOSD found that painful tonic spasms are more common in aquaporin-4 (AQP-4) antibody-positive than myelin oligodendrocyte glycoprotein (MOG) antibody-positive cases [80].

The pathophysiology of paroxysmal tonic spasms in MS or NMOSD remains unclear. Osterman and Westerberg proposed that ephaptic transmission between demyelinated axons [67] might be responsible. Alteration in supraspinal control might lead to a reduction in inhibitory spinal interneuron output, resulting in hyperexcitation of the alpha-motor neuron. Paroxysmal tonic spasms in MS or NMOSD are typically treated with low-dose carbamazepine [67, 81] or acetazolamide [74, 82]. Gabapentin has also been reported to be effective [83].

### Stiff person syndrome (SPS) and stiff limb syndrome (SLS)

Classic SPS is characterized by stiffness of the trunk and limb muscles. Axial musculature is usually involved, producing a classic hyperlordotic, dromedary gait. Stiff limb syndrome (SLS) is a rare form of SPS in which one limb is involved (Additional file 1: Video segment 1). SPS affects women disproportionately [84]. The electrophysiologic hallmark of SPS and SLS is continuous motor unit activity (CMUA) that persists even when the patient tries to relax [85]. High titers of glutamic acid decarboxylase (GAD) antibodies are found in 80 % of SPS [86]. GAD antibodies are not specific for SPS, as they may also be present in type I diabetes, autoimmune cerebellar ataxia, and autoimmunue polyglandular syndrome [87], either with or without SPS. Amphiphysin antibodies occur less commonly than GAD antibodies, and when present may signify an underlying malignancy [88–93]. While GAD and amphiphysin are proteins in the presynaptic terminal of inhibitory neurons, other antibodies to proteins at postsynaptic sites (GABA_A_ and glycine receptors) have also been reported in SPS [94–96]. Glycine receptor antibodies are not only associated with progressive encephalomyelitis with rigidity and myoclonus (PERM), but also classic and variant SPS, in both GAD antibody-positive and negative cases [96, 97].

Spinal and supraspinal pathways are important in SPS. Floeter and colleagues demonstrated abnormalities not only in spinal GABAergic circuits but also in spinal glycinergic inhibitory circuits [98]. The sensorimotor cortex [99] and motor and premotor cortices also likely play a role [100, 101]. Electrophysiologic abnormalities in the brainstem similar to hyperekplexia have also been demonstrated in SPS [102].

The diagnosis of SPS and SLS rests on the history, exam, comfirmation by physiology, and serology. Malignancy should be excluded, especially in SLS. Immunosuppressive therapy is the cornerstone of treatment, including intravenous steroids, IVIg or plasma exchange [91], although formal evidence-based guidelines regarding treatment are still lacking. For symptomatic treatment, oral pharmacologic agents include benzodiazepines, especially diazepam, and baclofen [85]. In cases where symptoms remain poorly controlled, intrathecal baclofen or spinal cord stimulation may be considered [103].

#### Progressive encephalomyelitis with rigidity and myoclonus (PERM)

PERM is considered to be a variant of SPS (“stiff-person-plus” syndrome) or a form of brainstem myoclonus [104]. It has been proposed to originate from the brainstem and spinal cord [104], and is thus not a pure SGMD. However, we include it in this review because of the clinical overlap with SPS and similarity in treatment approach. In the spinal cord, loss of spinal inhibitory interneurons leads to excess excitation of alpha-motor neurons, similar to SPS [105]. PERM was historically termed “spinal neuronitis” due to the finding of degeneration of long descending motor tracts [104], however cranial involvement and antibodies to glycine receptors [106] support a brainstem origin as well.

PERM is often rapidly progressive and typically involves the trunk or lower limbs. Myoclonus is often present, hence the term “stiff-person-plus” or “jerking SPS” [107]. Myoclonus typically involves the trunk or limbs as well as cranial-nerve innervated muscles, and is frequently stimulus-sensitive. Other brainstem signs such as nystagmus, oculomotor abnormalities, dysarthria, and dysphagia can be present, as well as cerebellar ataxia [108, 109], cognitive impairment, encephalopathy and seizure [106, 110, 111]. Dipeptidyl-peptidase-like protein-6 (DPPX) [108] and glycine receptor antibodies [96, 106, 111, 112] may be present, in addition to the previously mentioned anti-GAD antibodies [113]. The diagnostic work-up in patients with PERM includes imaging of the brainstem and spinal cord to rule out structural lesions, cerebrospinal fluid exam to look for infectious, parainfectious, paraneoplastic [114, 115] and autoimmune etiologies, as well as anti-GAD and glycine receptor antibodies in serum and cerebrospinal fluid. Evidence-based recommendations are lacking, but immunosuppressive therapies including steroids, IVIg, plasma exchange and others agents such as mycophenolate mofetil or azathioprine have been used. [106, 108, 110, 112].

### Movements in brain death

Movements in brain death are in fact normal findings and are not rare. They may be classified as *automatisms*, where movements occur spontaneously without stimuli, and *reflexes*, where movements occur after stimuli [116]. The latency between onset of brain death and the occurrence of movements varies from minutes to days [116, 117]. Spittler and colleagues proposed that this latency could be attributed to spinal shock. More than 40 types of movements have been described; most involve the trunk or limbs, however some involve cranial regions such as facial myokymia, eyelid opening and tongue myoclonus [118, 119].

Examples of automatisms include tonic arm flexion, abdominal contraction, knee, hip and leg flexion, and undulating toe movements [116, 120]. Reflex movements may occur after muscle stretch (muscle stretch reflexes), or stimuli to skin or deeper tissue (cutaneo-muscular reflexes). The reflexes can involve only one (monosegmental reflexes) or multiple segments (oligo- or polysegmental reflexes) of the spinal cord. Spittler and colleagues described 31 spinal reflexes [117], including muscle stretch reflexes of biceps, triceps, brachioradialis, finger flexion after biceps stretching and cremasteric reflexes. Neck flexion was reported to trigger movements such as shoulder protrusion, abdominal contraction, hip flexion and adduction or abdominal contraction. Movements following finger pinching included shoulder protrusion, abdominal contraction or arm/finger flexion. Sole stimulation, as in testing for the Babinski sign, led to hip and/or knee flexion or toe flexion [121]. Perhaps the most dramatic reflex is the Lazarus sign, in which both elbows and hands flex towards the chin and the patient may appear to arise from the bed [122, 123]. It can occur spontaneously (automatism) or as a reflex after stimuli such as during apnea testing or neck flexion. It is important for clinicians to recognize automatisms and reflexes so that the diagnosis of brain death is not distracted by their presence, and the patient’s family is appropriately reassured. For video examples, please refer to McNair and Meador [121], and Bueri et al. [122].

The movements of brain death are thought to be generated by the spinal cord. Several mechanisms have been proposed, including disinhibition of anterior horn cells disconnected from supraspinal control [116], and release of more phylogenetically primitive patterns of movement due to decoupling of the spinal cord from the neocortex and brainstem [117]. Interestingly, automatic stepping has been reported in patients prior to the declaration of brain death when the brainstem was compromised from central herniation [124]. In cats transected at the rostral margin of the superior colliculi, animals demonstrated spontaneous walking. The stepping reflex also occurs in infants before 6 weeks of age when they are held with feet placed on a flat surface. Collectively, these phenomena support the idea of a central pattern generator for locomotion within the spinal cord that may be responsible for spinal automatisms when it is disconnected from descending inhibitory influences.

### Painful legs-moving toes syndrome (PLMT)

Although named PLMT, this syndrome can be painless or even involve upper extremities, (painless legs-moving toes or painful arms [or hands]-moving fingers syndrome [125, 126]). The movements of toes or fingers are slow, writhing, typically 1–2 Hz, occurring in vertical (flexion/extension) and/or horizontal (abduction/adduction) planes (Additional file 3: Video segment 3). Involuntary movements of toes or fingers in a *pure* horizontal plane is virtually pathognomonic of the condition. Initially one limb may be involved, but contralateral spread can occur [127]. Pain is often the most debilitating feature of PLMT, and treatments such as gabapentin or pregabalin may be helpful [128]. Spinal cord stimulation has been reported to improve the movements [129]. PLMT may occur after nerve root, plexus or peripheral nerve lesions [127], and less commonly with spinal cord lesions or compression [127, 130]. The pathophysiology of PLMT remains unknown. One proposal included peripheral nerve pathology triggering central spinal and/or supraspinal reorganization [127, 131, 132]. Pain, possibly attributed to central sensitization, and the complexity of the involuntary movements support a central rather than peripheral etiology [127].

PLMT is difficult to treat. Studies or reports have been conducted on a small scale. It is however important to look for and treat an underlying peripheral neuropathy. In addition to gabapentin, pregabalin and spinal cord stimulation as mentioned above, various treatments reported to be effective include baclofen, carbamazepine [127, 133], benzodiazepines such as clonazepam, tricyclic antidepressants [134], adenosine [135], sympathetic blockade [127, 136], epidural block [137], transcutaneous electrical nerve stimulation [138] and botulinum toxin injection [139–141].

## Conclusion

SGMDs are a unique group of movement disorders, sharing many features. We hope that our review will help clinicians care for these challenging patients.

## Video segments

### Video segment 1

Additional file 1: Video segment 1 (https://drive.google.com/open?id=0BxN303l61fkBdlVyT2VJU2I1aEE) demonstrates the phenomenology of spinal segmental myoclonus, priopriospinal myoclonus (PSM) and stiff limb syndrome. *Patient 1* is a 50-year-old woman with a 3-year history of spinal segmental myoclonus affecting the lower back and abdomen. Examination revealed symmetric volleys of myoclonic jerks in abdominal muscles, worsened when she lifted her legs against resistance. A rectus jerk could be elicited by tapping the patellar reflex with sufficient force, illustrating stimulus-sensitivity, unusual for this disorder. *Patient 2*, a 31-year-old woman, developed spinal segmental myoclonus several weeks after a severe viral upper respiratory infection. Myoclonic jerks of the thoracic abdominal muscles were rapid and symmetric. Movements interfered with her ability to provide diaphragmatic support for speech or sustained vocalization. *Patient 3* demonstrated spinal segmental myoclonus of the thoracic abdominal muscles, worse on leaning to the right. The next three patients demonstrate classic phenomenology of PSM. Of note, the diagnosis of PSM in our patients was made on clincial grounds. The more specific diagnosis of organic or psychogenic PSM cannot be confirmed due to unavailibility of electrophysiologic study. *Patient 4* is a 62-year-old woman who developed propriospinal myoclonus in her upper legs, buttocks and lower torso. Movements occurred with sitting and lying down, disappeared on standing, and persisted in sleep (by report). A charactersitic delay in reflex-induced myoclonic jerks was evident bilaterally, with both the biceps and knee jerks. *Patient 5* is a 64-year-old woman with a history of thoracic spine surgery at the T7-8 level, with a 13-year history of priopriospinal myoclonus. Abdominal jerks coalesce in the video to produce continuous abdominal spasms. Biceps and triceps reflexes trigger a time-delayed jerk that also flexes the trunk. *Patient 6* demonstrates propriospinal myoclonic jerks of the trunk on standing which were initially dismissed as psychogenic. A characteristic time-delayed jerk is evident with biceps and patellar reflexes. The work up of Patients 1–6 included spinal magnetic resonance imaging which was negative for structural lesions in the spinal cord. Clonazepam, valproic acid and/or levetiracetam were employed in these patients with variable improvement (not shown in the video). *Patient 7* developed stiffness and cramping of his left leg with walking. His medical history was notable for juvenile diabetes, and anti-GAD antibody was markedly positive. Continuous motor unit firing on EMG confirmed the diagnosis of stiff limb syndrome (SLS). Treatment with intravenous immunoglobulin and diazepam dramatically improved his symptoms.

### Video segment 2

Additional file 2: Video segment 2 (https://drive.google.com/open?id=0BxN303l61fkBYnBMeVFqazNib2s) demonstrates the rich phenomenology of orthostatic tremor (OT) in five patients. *Patient 1* is an 83-year-old man who presented with a 10-year history of difficulty standing while shaving or waiting for a train. Examination revealed OT, visible and palpable in the legs, as well as sense of imbalance triggered by standing and immediately relieved by walking. *Patient 2* is a 75-year-old woman with a thoracic arachnoid cyst and a 3-year history of OT. Symptoms were triggered by standing, relieved by walking, and recurred even as she briefly stopped to turn. The movements may be secondary OT. We cannot prove they are the result of the arachnoid cyst, but they started after the cyst was recognized. *Patient 3*, an 86-year-old man with a 1-year history of significant OT, demonstrates the marked benefit he obtains from peripheral sensory input (gently holding the examiner’s hand, or touching the wall with his hand). The video mimics how he uses this sensory input to ambulate in his home, walking while guiding the palm or even the dorsum of his hand along the wall. *Patient 4*, a 76-year-old woman with a 6-year history of severe OT, had already developed gait fearfulness by the time of her evaluation. Examination in the office revealed OT which improved instantly by grasping the examiner’s hand or even one finger. She would not allow the examiner to let go of her hands to stand unassisted. Treatment with clonazepam 0.5 mg twice daily markedly improved her OT, allowing her to walk unassisted (not shown on video). *Patient 5*, a 70-year-old woman with a 3-year history of OT, could no longer walk without a rolling walker. Examination demonstrated OT that would occur within 5 seconds of standing and was relieved by touching the table. Her walking was impaired although she was able to ambulate with the sensory input of holding one finger of the examiner’s hand. Treatment with clonazepam 0.5 mg twice daily allowed her to stand and walk unassisted (demonstrated on video).

### Video segment 3

Additional file 3: Video segment 3 (https://drive.google.com/open?id=0BxN303l61fkBX0dGWi1WR3gtZGc) demonstrates the phenomenology of painful legs-moving toes (PLMT) and painful hands-moving fingers (PHMF) syndromes. *Patient 1* presented with a 3-year history of involuntary movements of the toes, observed even in deep sleep. Movements were accompanied by pain that was refractory to medication. Examination revealed bilateral undulating movements of the toes in both flexion/extension and the lateral plane. *Patient 2* presented with involuntary movements of the left toes which were annoying but not painful. Examination revealed undulating lateral movements of all five toes, consistent with painless, unilateral PLMT. *Patient 3* demonstrates similar movements affecting the fingers of the left hand, accompanied by pain that persisted in sleep—PHMF *Patient 4*, a 53-year-old man with a 20-year history of pain and involuntary movements of the left hand, demonstrates a similar phenotype of PHMF. Side to side undulating movements of the third, fourth and fifth fingers were disabling and refractory to treatment.

### Consent statement

Written informed consent was obtained from the patients for publication of all video segments. A copy of the written consent is available for review by the Editor-in-Chief of this journal.

## Additional files

Additional file 1:
**Video segment 1 demonstrates the phenomenology of spinal segmental myoclonus, priopriospinal myoclonus (PSM) and stiff limb syndrome.** See section on Video segments for more details. (MP4 610968 kb) Additional file 2:
**Video segment 2 demonstrates the rich phenomenology of orthostatic tremor (OT) in five patients.** See section on Video segments for more details. (MP4 503870 kb) Additional file 3:
**Video segment 3 demonstrates the phenomenology of painful legs-moving toes (PLMT) and painful hands-moving fingers (PHMF) syndromes.** See section on Video segments for more details. (MP4 211600 kb)
