# Publisher’s Erratum: Spinal-generated movement disorders: a clinical review

**DOI:** 10.1186/s40734-016-0042-y

**Published:** 2016-11-22

**Authors:** Pichet Termsarasab, Thananan Thammongkolchai, Steven J. Frucht

**Affiliations:** 1 Department of Neurology, Movement Disorder Division, Icahn School of Medicine at Mount Sinai, New York, USA; 2 Department of Medicine, Neurology Division, Faculty of Medicine, Siriraj Hospital, Mahidol University, Bangkok, Thailand; 3 Department of Neurology, University Hospitals Case Medical Center, Cleveland, USA

## Erratum

Following publication of the original article [1], it was noticed that the video files for this article (included as supplementary material) were not functioning correctly. Please see below for the corrected files:

## Additional files

Additional file 1: Video segment 1 demonstrates the phenomenology of spinal segmental myoclonus, priopriospinal myoclonus (PSM) and stiff limb syndrome. See section on Video segments for more details.
Additional file 2: Video segment 2 demonstrates the rich phenomenology of orthostatic tremor (OT) in five patients. See section on Video segments for more details.
Additional file 3: Video segment 3 demonstrates the phenomenology of painful legs-moving toes (PLMT) and painful hands-moving fingers (PHMF) syndromes. See section on Video segments for more details.
